# Limited impact of a top-down approach to improve enhanced recovery programme in French university hospitals: a before-after retrospective survey

**DOI:** 10.1186/s13741-021-00200-9

**Published:** 2021-09-06

**Authors:** Hakim Harkouk, Perrine Capmas, Nawal Derridj, Anissa Belbachir, Lionelle Nkam, Philippe Aegerter, Eva Battaglia, Laure Tharel, Dominique Fletcher

**Affiliations:** 1 Anaesthesia and Intensive Care Department, Ambroise Paré Hospital, APHP, 9 avenue Charles de Gaulle, 92100 Boulogne-Billancourt, France; 2 Université Paris-Saclay, UVSQ, Inserm, LPPD, 92100 Boulogne, France; 3 Obstetric Gynecology Department, Bicêtre Hospital, APHP, Le Kremlin-Bicêtre, France; 4 Clinical Research Unit, Ambroise Paré Hospital, APHP, Boulogne-Billancourt, France; 5 Anaesthesia and Intensive Care Department, Cochin Hospital, APHP, Paris, France; 6 GIRCI-IDF, Cellule Méthodologie, Paris, France; 7 Université Paris-Saclay, UVSQ, Inserm, Équipe d’Épidémiologie respiratoire intégrative, CESP - Centre de recherche en Epidémiologie et Santé des Populations U1018 INSERM UPS UVSQ, 94807 Villejuif, France; 8 Direction de la Politique et de la Transformation, Assistance Publique Hôpitaux de Paris, Paris, France

**Keywords:** Surgery, Enhanced recovery, Length of stay, Post-operative complications

## Abstract

**Background:**

Enhanced recovery programme (ERP) after surgery needs development in Assistance Publique Hôpitaux de Paris (APHP).

**Methods:**

A retrospective before-and-after study was performed in 2015 and 2017 on three surgical models (total knee arthroplasty (TKA), colectomy and hysterectomy) in 17 hospitals including 29 surgical departments. Data were collected in one control intervention (total hip arthroplasty (THA), gastrectomy and ovariectomy). In 2016, Massive Open Online Course on ERP and a day meeting information were developed by APHP. A national update on ERP was also organized by HAS and a regional professional partnership programme was started. Primary outcomes were length of stay (LOS) and complications after surgery. Data on ERP items were collected in the patients’ chart and in anaesthetist and surgeon interview. Seventy percent application rate reflects application of ERP procedure.

**Results:**

1321 patient’s files were analysed (812 in 2015 and 509 in 2017). The LOS (mean (SD)) is reduced by 1.6 day for TKA (2015, 8.7 (6.7) versus 7.1 (3.4) in 2017; p<0.001) but stable for colectomy and hysterectomy. Incidence of severe complications after surgery is unchanged in all types of surgical models. For TKA and hysterectomy respectively applied items of ERP (i.e. >70% application) increased respectively from 5 to 7 out of 17 and 16 in 2015 and 2017. For colectomy, they were stable at 6 out of 21 in 2015 and 2017. The mean application rates of ERP items stayed below 50% in all cases in 2017. The LOS was negatively correlated with ERP items’ application when data collected in 2015 and 2017 were analysed together.

**Conclusion:**

ERP application did not significantly improved between 2015 and 2017 for three surgical models after an institutional information and diffusion of recommendations in 29 surgical departments of seventeen French University hospitals underlining the limit of a top-down approach.

## Introduction

Enhanced recovery programme (ERP) for surgical patients is an approach for perioperative multimodal management of the patient aiming at a rapid restoration of previous physical and mental capacities allowing the reduction of perioperative morbidity. It also results in shorter hospital stays. This approach corresponds to a specific organization of care around patient-centred clinical paths. A recent meta-analysis confirms its effectiveness in reducing the length of stay and the overall rate of complications all specialties combined (Nicholson et al. 2014). However, several authors point out the difficulties or failures of the method. These guidelines remain difficult to adopt essentially because they require the simultaneous and complex involvement of all members of the perioperative team (Kahokehr et al. 2009). A Canadian team, having deployed an ERP on colonic surgery in more than 2 years in 15 academic hospitals, concludes to the importance of a demand that emerges from the field and the need for the cohesion of the institution (McLeod et al. 2015). However, other organizational aspects are to be credited with this approach: the benefits observed, in particular in terms of care load, would allow the movement to be maintained and there is a diffusion effect towards other interventions initially not concerned (Kahokehr et al. 2009).

In 2016, the French Haute Autorité de Santé has proposed an update in the form of an orientation report and a memo sheet encouraging the development of the ERP (HAS 2016), while the Assistance Publique Hôpitaux de Paris (APHP) was mobilizing as institution to develop ERP in 2016. The evaluation of dissemination and appropriation of multi-specialty recommendations in a large group of teaching hospital would be a first in France. The use of innovative means such as an open online training site (MOOC, Massive Open Online Course) can make it easier to reach all the stakeholders concerned to promote generalization of organizational innovations.

Thus, the primary objective of this study is to measure the effects of this top-down approach on the length of hospital stays and on safety of the implementation of ERP, throughout several hospitals in the APHP institution.

## Methodology

### Population

Inclusion criteria: adult patient, without opposition to the extraction of data from her/his file and the follow-up on day 30, scheduled for one surgical model interventions or a control intervention. The following interventions were studied as ERP models since specific guidelines exist for ERP in these models, and they are frequent (≥ 600 annual stays at the APHP) and carried out in around ten different services (which will allow an analysis of the determinants at the service level). We chose total knee arthroplasty (approximately 1500 interventions per year on 12 sites, 10 of which are active (i.e. > 30 interventions/year)), left colectomy for cancer or non-cancer pathology (600 annual interventions on 17 sites, 9 of which have an activity > 30 interventions/year) and hysterectomy (approximately 570 procedures per year on 16 sites, 12 of which have an activity > 30 procedures/year). This audit covered both the target intervention (ERP interventions) and control interventions of the same severity not specifically targeted by the ERP approach (i.e. having not benefited from specific clinical paths). Three other surgical models were analysed as control to observe spontaneous evolution of medical process: total hip arthroplasty for orthopaedic surgery, gastrectomy for visceral surgery and ovariectomy for gynaecological surgery.

Each selected centre had more than 30 interventions per year for the selected surgical model and 30 randomly selected files were analysed per centre (20 files for ERP interventions and 10 for control interventions).

Non-inclusion criteria: none.

### Intervention

The comparison scheme was “before-and-after” type for the two 2015–2017 periods comparison. We consider the year 2015 as a “reference” year and the year 2017 as a year of full implementation, 2016 being for launching.

Between the two evaluations period, an institutional awareness-raising phase on ERP in 2016 was composed of 5 steps: (1) 1 day of sensibilization on ERP in the institution in April 2016 with a meeting of 200 health care providers and institutional stake holders; (2) development of a Massive Open Online Course on ERP in July 2017 followed by 700 participants; (3) diffusion of national update on ERP by Haute Autorité de Santé in June 2016 to all surgical and anaesthesia departments of APHP (4); (4) institutional access to *GRACE* group in 2016 for all surgical and anaesthesiology departments offering access to guidelines, scientific literature and possibility to organize survey and benchmarking (5); and (5) preparation of participation to a regional programme organized by Regional Health Agency (Agence Régionale de Santé) offering inter institutional collaboration on ERP. This was the initiative of ten surgical departments out of 29 involved in the survey participated in 2017 to the Regional Health Agency training programme offering inter institutional collaboration on ERP. These centres were involved as learning centres or teaching expert based on the existence of ERP protocols and previous evaluation with GRACE group. Four participate in orthopaedic surgery (2 experts, 2 learning centres), 3 in visceral surgery (1 expert, 2 learning) and 3 in gynaecological surgery (1 expert, 2 learning).

### Outcomes

#### Patients

The main demographic (age, sex), clinical (main pathology, comorbidities, ASA score, autonomy) and sociologic (living alone) characteristics were collected simultaneously. The Charlson score was calculated for all patients.

#### Length of stay, complications and rehospitalization

Patients’ files were used to obtain duration of stay and incidence of complications after surgery. A list of eight complications was used for identification in the patient’s file (i.e. transfer to intensive care unit, new surgery, bleeding, infection requiring antibiotic, pulmonary embolism or venous thrombosis, allergy, aggravation of existing medical condition, other). It was defined in the patient’s chart whether this complication has prolonged the hospital stay or if a new hospitalization in the same institution occurred within 30 days after surgery.

#### Patient management and ERP

For each ERP model intervention, a specific grid was designed based on existing ERP guidelines in 2016 for each surgery to collect data describing the patient management process pre, per and postoperatively (Alfonsi et al. 2014). The total ERP item number was 17 for TKA, 21 for colectomy and 16 for hysterectomy. The cut-off value of 70% for ERP item application (i.e. 70% of a particular item is applied on the patient population) was considered as reflecting sufficient appropriation (Gustafsson et al. 2011).

#### Interview of professionals

It was performed when research assistant visits the hospital for data collection with a representative of surgical and anaesthesiology department to describe level of development of ERP, resources issue and difficulties. Concerning services, the characteristics collected concerned structure (equipment) and resource (personnel, qualification) data, commitment to quality improvement approach (Morbi mortality review; quality professional improvement) and existing clinical pathways. The same independent dedicated staff trained to data collection, collected all data and performed interview in each centre.

### Statistical analysis

Primary outcome was the length of stay of the different surgical models selected, over 2 periods of 1 year, before (year 2015) then after (year 2017) the institutional awareness-raising phase on ERP. The secondary objectives were effects on the length of hospital stays in each centre following an awareness phase on the practice of ERP; evaluation of the conformity of practices with regard to ERP before and after awareness-raising phase; and evaluation of the incidence of 30 days’ post-intervention complications, in each intervention, before and after the implementation of the program.

We postulated that the implementation of the ERP would modify the compliance rate from 60% of non-compliant files “before” to 40% of non-compliant files “after”, then requiring, for a two-sided alpha risk of 5% and 80% power, 2 groups of 110 files, i.e. 220 files in total (110 before and 110 after) per model intervention. For LOS, we target a minimal effect of 1.1 day reduction (Nicholson et al. 2014), since SD for LOS is around 3 (Gianotti et al. 2014), for a two-sided alpha risk of 5% and 80% power, 2 groups of 118 files, i.e. total of 236 files per model intervention.

However, there is probably a cluster effect at hospital level, all the interventions of a model being supported by a single anaesthesia service and a single surgical team, and therefore, the observations of the same hospital tend to resemble each other and provide less information than the same number of independent observations. Considering the literature on cluster effect for process-related variables, we retained a 0.02 ICC (Fletcher et al. 2008). Then, starting from 25 files on average per hospital and model, the design effect is around 1.5, and 380 files must be studied per model intervention (190 before and 190 after) to obtain the same information amount. The number of “control” interventions is halved.

All comparisons were made with the chi-square test or Fisher test for discrete variable and t-test or Wilcoxon rank-sum test for continuous variable between 2015 and 2017 periods for the whole population and each centre for control and ERP surgical models.

A linear mixed model was used to identify the variables related to LOS among patient’s characteristics, by taking into account the existing correlation between files from the same surgical department.

Missing data were analysed depending on the type of item and best available interpretation. Most of the items that were not available in the patient’s chart were considered as absent and non-performed.

## Results

### Data collected

Seventeen hospitals participated with a total of 29 surgical and 17 anaesthesia departments involved. No data were collected in 2017 in 11 surgical departments of 6 hospitals. A total of 1321 patient’s files were collected in 18 surgical departments (812 in 2015 and 509 in 2017; Table 1). Data were collected for “before” between November 2017 and June 2018 and for “after” between December 2018 and February 2020.

**Table 1:** Patients included

Type of surgery	2015	2017
Total knee arthroplasty	195	137
Colectomy	163	96
Hysterectomy	190	120
Total hip arthroplasty	99	66
Gastrectomy	69	32
Ovariectomy	96	58
Total	**812**	**509**

### Demographics and patients’ characteristics

Data are listed in Table 2. The patients are younger in 2017 only after TKA. In most surgical models, patients are living alone more frequently in 2017.

**Table 2:** Patient’s characteristics

Patient’s characteristics	Age (years) 2015/2017/p*	Sex (male: n/%) 2015/2017/p	ASA score (1, 2, 3, 4; n/%) 2015/2017/p	Charlson (2015/2017/p)	Live style: not living alone (n/%) 2015/2017/p
TKA (*n* = 195/137)	70.9 (10.6)/67.7 (11.7) *P* = 0.004	61 (31.3)/36 (26.3) NS	1 = 14 (7.2)/11 (8.0) 2 = 113 (57.9)/94 (68.6) 3 = 48 (24.6)/16 (11.7) *P* = 0.034	0 = 5 (2.6)/6 (4.4) 1 = 12 (6.2)/17 (12.4) 2–7 = 178 (91.3)/114 (83.2) *P* = 0.084	98 (50.3)/39 (28.5) *P* < 0.0001
Colectomy (*n* = 163/96)	61.5 (14.9)/60.6 (14.4) NS	89 (54.6)/47 (49.0) NS	1 = 26 (16.0)/26 (27.1) 2 = 94 (57.7)/52 (54.2) 3: 25 (15.3)/12 (12.5) NS	0 = 36 (22.1)/22 (22.9) 1 = 27 (16.6)/22 (22.9) 2–7 = 100 (61.3)/52 (54.2) P = 0.399	93 (57.1)/34 (35.4) *P* < 0.0001
Hysterectomy (*n* = 190/120)	52.5 (11.6)/51.4 (11) NS	-	1 = 58 (30.5)/38 (31.7) 2 = 92 (48.4)/55 (45.8) 3 = 5 (2.6)/7 (5.8) NS	0 = 99 (52.1)/69 (57.5) 1 = 44 (23.2)/24 (20.0) 2–7 = 47 (24.7)/27 (22.5) *P*=0.643	95 (50.0)/49 (40.8) *P* = 0.033
THA (*n* = 99/66)	68.4 (14.9)/66.4 (14.2) NS	35 (35.4)/27 (40.9) NS	1 = 15 (15.2)/10 (15.2) 2 = 53 (53.5)/31 (47.0) 3 = 21 (21.2)/20 (30.3) NS	0 = 11 (11.1)/8 (12.1) 1 = 16 (16.2)/13 (19.7) 2–7 = 72 (72.7)/45 (68.2) *P* = 0.806	51 (51.5)/20 (30.3) *P* < 0.0001
Gastrectomy (*n* = 69/32)	62.9 (14.6)/65.4 (15) NS	37 (53.6)/24 (75.0) NS	1 = 11 (15.9)/2 (6.2) 2 = 35 (50.7)/14 (43.8) 3 = 18 (26.1)/7 (21.9) P = 0.045	0 = 14 (20.3)/4 (12.5) 1 = 12 (17.4)/7 (21.9) 2–7 = 43 (62.3)/21 (65.6) *P* = 0.602	45 (65.2)/11 (34.4) *P* = 0.005
Ovariectomy (*n* = 96/58)	48.4 (15)/45.9 (17.6) NS	-	1 = 36 (37.5)/24 (41.4) 2 = 39 (40.6)/17 (29.3) 3 = 6 (6.2)/6 (10.3) NS	0 = 53 (55.2)/32 (55.2) 1 = 20 (20.8)/14 (24.1) 2–7 = 23 (24.0)/12 (20.7) *P* = 0.84	46 (47.9)/24 (41.4) NS

P*, Wilcoxon rank-sum tests

The main demographic (age, sex), clinical (main pathology, comorbidities, ASA score) and sociologic (living alone) characteristics were collected simultaneously. The Charlson score was calculated for all patients and high score (i.e. 2–7) compared with low score (i.e. 0 and 1)

### Length of stay and complications (Table 3)

In orthopaedic surgery, the length of stay (LOS) was reduced by 1.6 days (2015, 8.7 (6.7) versus 7.1 (3.4) in 2017; *p*<0.001). It was stable for colectomy and hysterectomy as in control groups (i.e. THA, gastrectomy and ovariectomy).

**Table 3:** Length of hospital stay and complications within 30 days after surgery

Type of surgery/outcome	Length of hospital stay (day) 2015/2017/p*	Incidence of complications within 30 days after surgery (n/%) 2015/2017/p	Prolongation of hospital stay (day) 2015/2017/p	New hospital admission within 30 days after surgery 2015/2017/p
TKA (*n* = 195/137)	8.7 (6.7)/7.1 (3.4) *p* < 0.001	17 (8.7%)/12 (8.8%) NS	11(5.6%)/1(0.7%) *p* = 0.003	11 (5.6%)/2 (1.5%) NS
Colectomy (*n* = 163/96)	10.5 (6.5)/13.8 (38.1) NS	18 (11%)/11(11.5%) NS	7 (4.3%)/8 (8.3%) NS	5 (3.1%)/5 (5.2%) NS
Hysterectomy (*n* = 190/120)	4.6 (2.6)/4.2 (2.3) NS	9 (4.7%)/4 (3.3%) NS	6 (3.2%)/3 (2.5%) NS	6 (3.2%)/2 (1.7%) NS
THA (*n* = 99/66)	8.2 (4.1)/8.7 (15.5) *p* = 0.011	11 (11.1%)/1 (1.5%) *p* = 0.029	5 (5.1%)/1 (1.5%) NS	3 (3.0%)/2 (3.0%) NS
Gastrectomy (*n* = 69/32)	17.7 (17)/15.7 (8.6) NS	22 (31.9%)/9 (28.1%) NS	15 (21.7%)/6 (18.8%) NS	7 (10.1%)/3 (9.4%) NS
Ovariectomy (*n* = 96/58)	5.5 (24.2)/3.7 (4.6) NS	2 (2.1%)/3 (5.2%) NS	2 (2.1%)/3 (5.2%) NS	1 (1.0%)/1 (1.7%) NS

P*, Wilcoxon rank-sum tests

The incidence of complications within the first month was unchanged in all types of surgical models except in THA patients (11.1% in 2015 versus 1.5% in 2017, p=0.029). The prolongation of hospital stay related to complications was reduced in frequency after TKA.

### Patient management and ERP compliance

Some ERP items were detailed in the results but treated as one single item (i.e. oral or subcutaneous thromboprophylaxis; technique of regional anaesthesia for pain control; less invasive surgical technique).

#### Orthopaedic surgery (Fig. 1)

For TKA, the regional anaesthesia techniques (i.e. femoral block, infiltration and adductor canal block) evolved over time and the three techniques were considered as appropriate and cumulated in one item. The applied items of ERP (i.e. >70% application) increased from 5 to 7 out of 17 between 2015 and 2017 with improvement on intraoperative use of dexamethasone and early oral nutrition. The evolution for ERP compliance was similar for THA (data not shown).

**Fig. 1:**
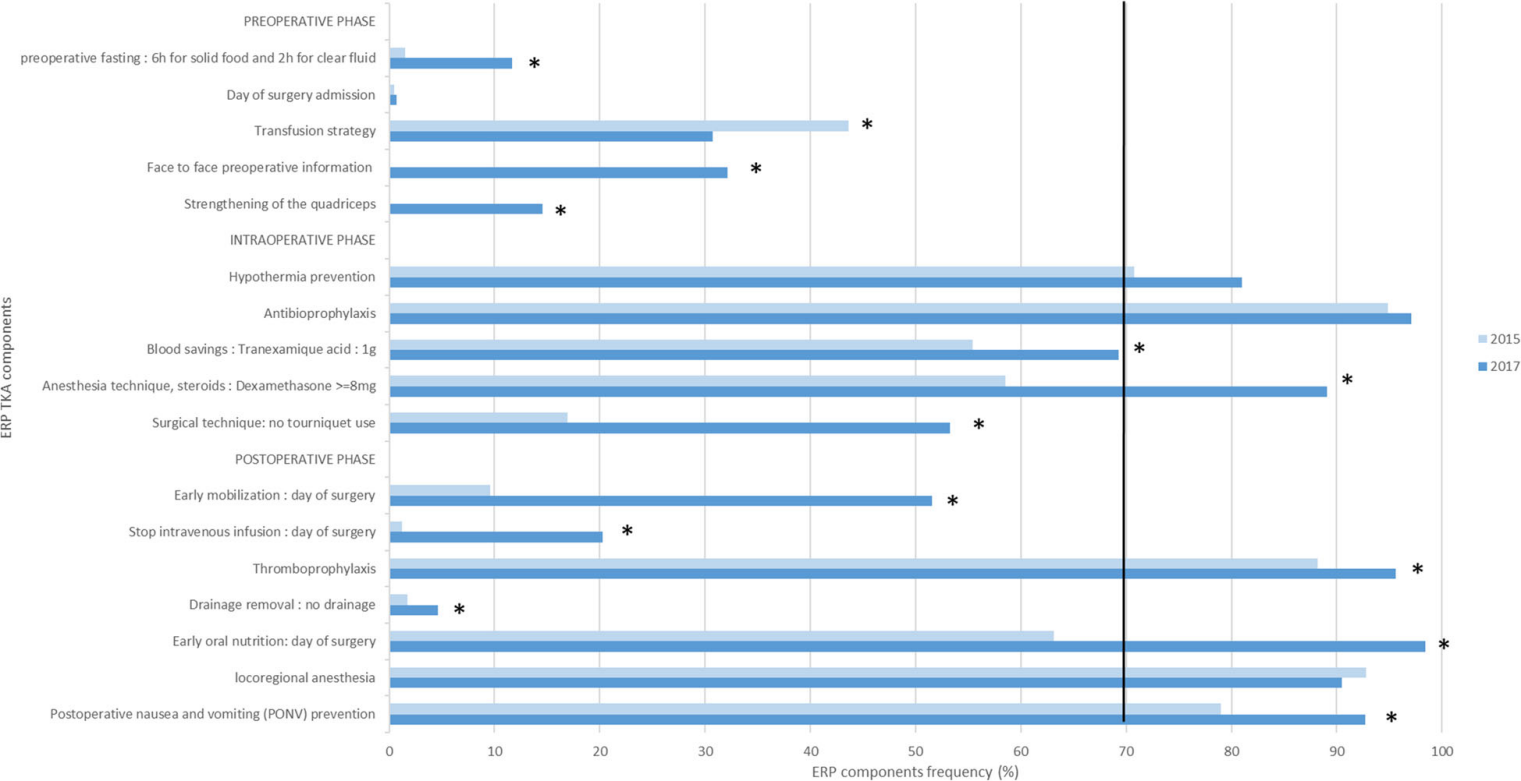
ERP components adherence in TKA patients in 2015 and 2017

#### Visceral surgery (Fig. 2)

For colectomy, applied items of ERP (i.e. >70% application) were stable at 6 out of 21 with no improvement between 2015 and 2017. The evolution for ERP compliance was similar for gastrectomy (data not shown).

**Fig. 2:**
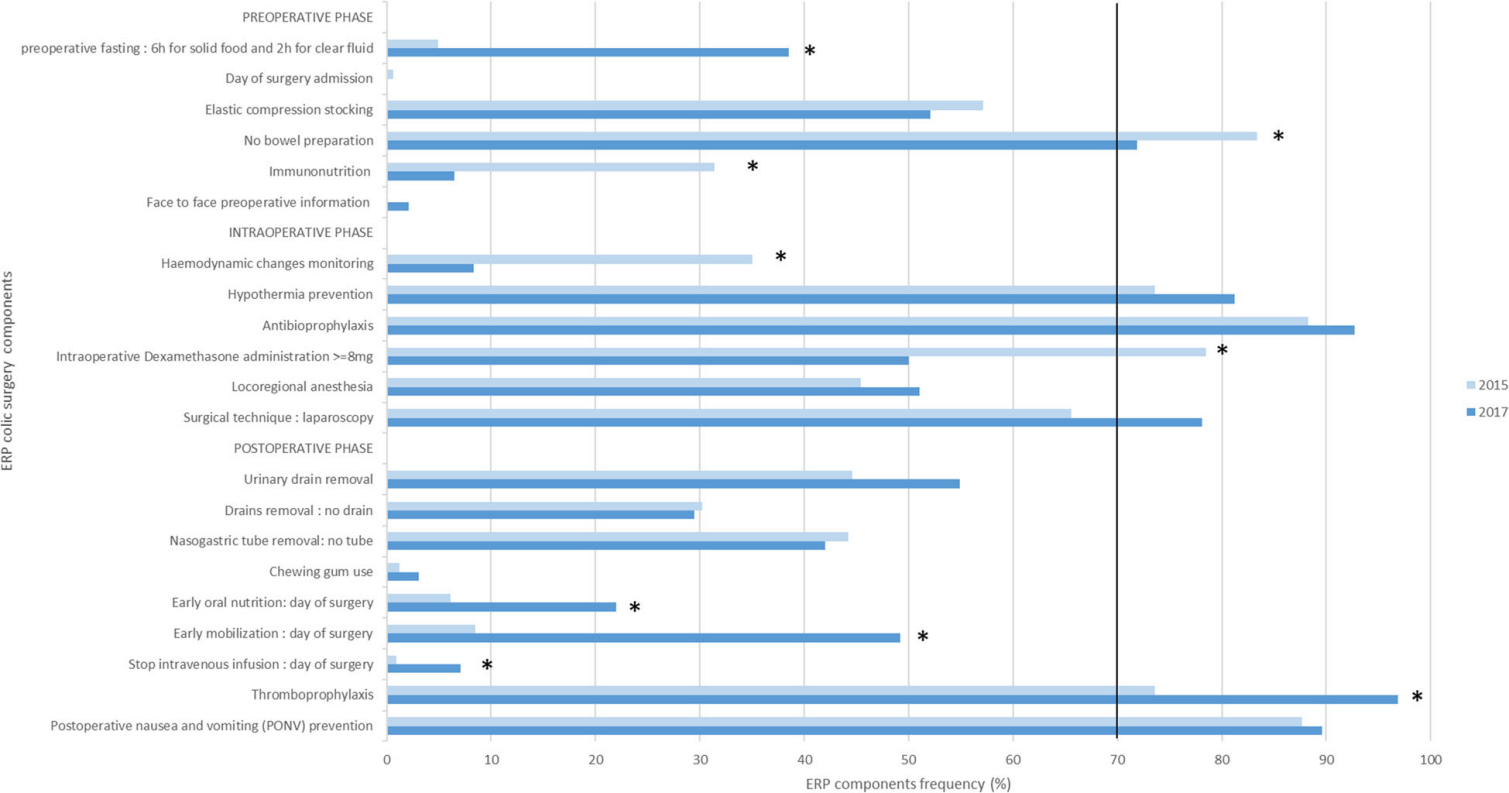
Comparison of frequency of ERP items application for colectomy in 2015 and 2017

#### Gynaecological surgery (Fig. 3)

For hysterectomy, applied items of ERP (i.e. >70% application) increased from 5 to 7 out of 16 between 2015 and 2017 with improvement on elastic compression stocking and vaginal packing. The evolution for ERP compliance was similar for ovariectomy (data not shown).

**Fig. 3:**
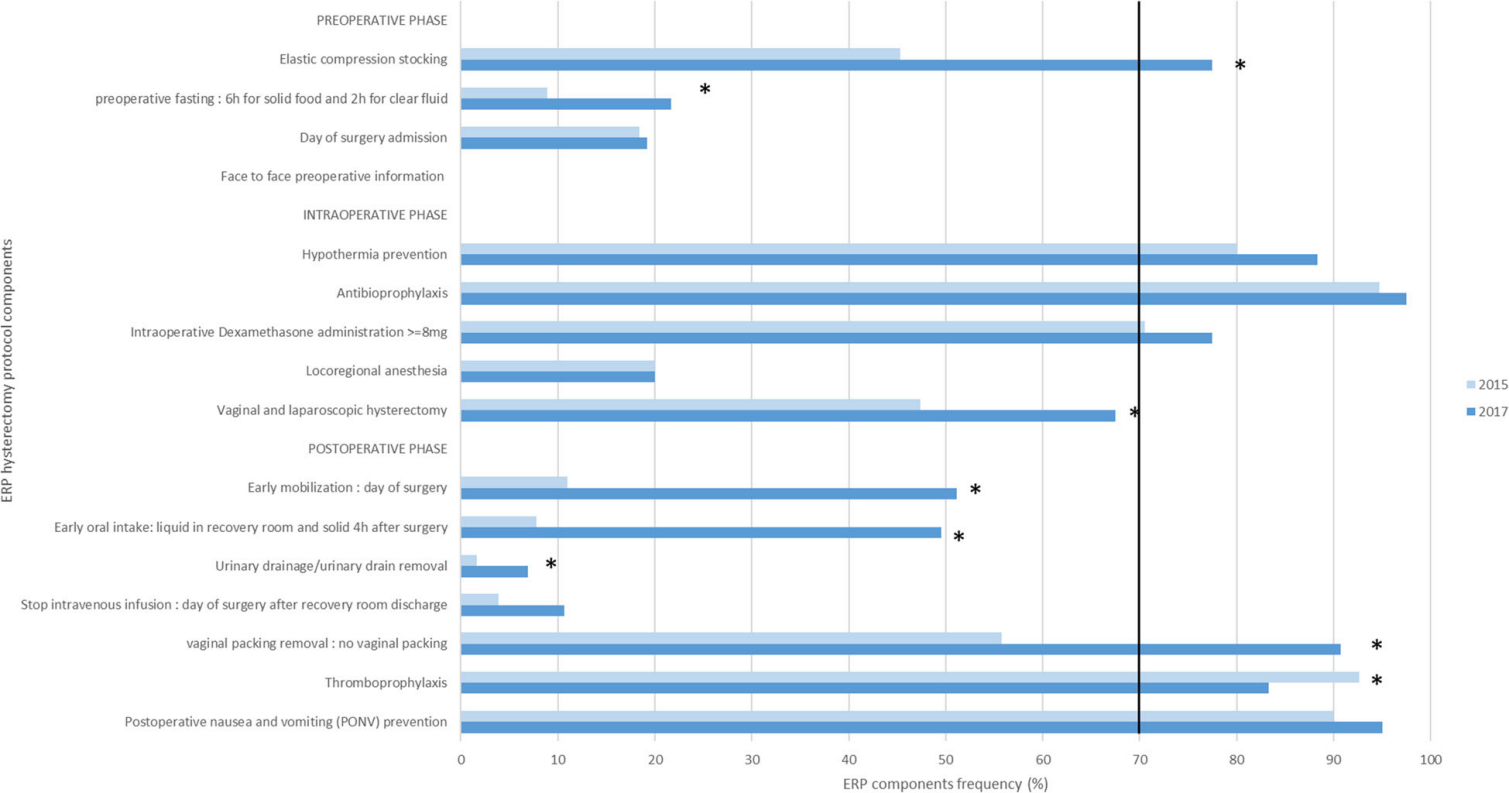
Comparison of frequency of ERP items application for hysterectomy in 2015 and 2017

#### Individual trajectory of different structures

All centres with data collected at 2015 and 2017 progressed in ERP between 2015 and 2017 for TKA (7/7) and hysterectomy (6/6) with no significant difference between centres. Evolution was mixed for colectomy with some centres reducing their application (2/5) and some improving (3/5) with no significant difference between centres. For the 6 learning centres participating to the Regional Health Agency training programme, the trajectory was not significantly different from other centres (p>0.99). The single expert centre in gynaecological surgery has the best performance on 2017; the 2 expert centres in orthopaedic surgery were the second and third best centres in 2017; in visceral surgery, the only expert centre did not collect data in 2017.

### Correlation between patient management and outcome (Fig. 4)

All individual data of both periods before and after were analysed for relation between individual LOS and number of ERP items applied. There is a significant correlation between LOS reduction with increased application of ERP for TKA, colectomy and hysterectomy (Spearman test; p value and correlation coefficient: TKA 0.00003 and − 0.202; colectomy < 0.001 and − 0.232; hysterectomy 0.0018 and − 0.182 respectively). When grouping in three categories according to a previous publication describing a dose-response relationship between various levels of ERP item application and LOS (Gustafsson et al. 2011) (i.e. ERP items application < 50%; 50–70%, > 70%), there is a significant relation for TKA (*p*=0.002) and hysterectomy (*p*=0.002; Kruskal-Wallis test).

**Fig. 4:**
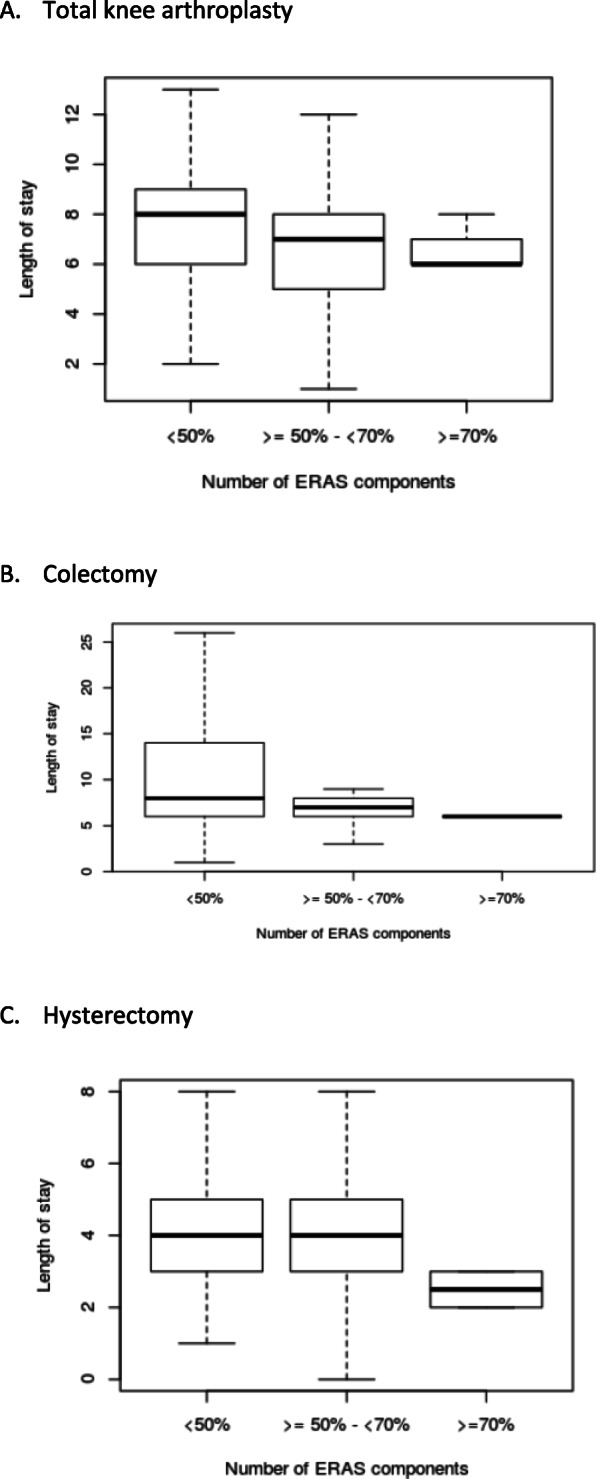
Evolution of length of stay depending of the percentage of ERP items application. **A** Total knee arthroplasty. **B** Colectomy. **C** Hysterectomy

### Structural and organizational evaluation with interview of professionals

The results in the three surgical models were similar with declaration of increased implication of health care providers in ERP between 2015 and 2017. However, compared to observed data in ERP application, the declarative information generally overestimates the level of performance. As an example, the rate of specific patient information on ERP was declared as 100% when maximal observed rate was 32% for TKA. The health care providers declare that they use specific indicators to monitor ERP evaluation in 50% of cases in 2015 and 100% in 2017. Despite institutional availability in all surgical and anaesthesiology departments, rate of GRACE survey tool was declared as persistently low (0% in 2015 and 14% in 2017). Frequently declared encountered difficulties are lack of adherence to the ER concept and lack of multidisciplinary communication and limited manpower, specifically ERP nurse coordinator. Organization of ERP was also limited by the difficulty to organize admission on same day, lack of available time to start ERP and patients’ reluctance.

## Discussion

This survey in twenty-nine surgical departments located in seventeen different university hospitals of Assistance Publique Hôpitaux de Paris (APHP) describes no clinically significant improvement in the development on ERP with a top-down approach. We observed a correlation between ERP items application and LOS.

### Top-down approach did not significantly reduce LOS and postoperative complications

We observe a significant reduction of LOS only after TKA, but no modification in the other surgical models and no reduction of postoperative complications incidence in any surgical model. This negative result reflects probably the inability to centrally decide the evolution of organization required at the institution level to improve ERP. In fact, we chose to start the implementation of ERP in our institution by a top-down approach which according to our survey, appears not clinically beneficial. In determinant factors involved in the ERP implementation, the multidisciplinary health care provider team mobilization and coordination is the corner stone for improvement (Slim et al. 2016). In our approach, these teams were indirectly motivated by improvement measures proposed in 2016 at the APHP institutional level. The MOOC was followed by 700 participants and the 1-day ERP sensitization meeting by 200 persons but it is still low compared to the size of the APHP institution with a total of 60,000 health care providers. Therefore, these improvement measures proposed in 2016 were probably insufficient to motivate and impulse change necessary for ERP application. In fact, qualitative evaluation of health care professionals’ perception of key of success for ERP implementation underlines multiple structuring mechanisms which need to be activated in parallel (Petit et al. 2021; Herbert et al. 2017). They combine appropriated evidence-based patient management protocols, team animation and leadership, questioning practice and adaptation, standardization of care, auto evaluation and multi-disciplinary communication (Petit et al. 2021; Herbert et al. 2017). We did not have sufficient resources to provide this personalized approach on a large scale. Our general top-down initial approach did not offer these crucial levers and it should have been coordinated with local multidisciplinary team mobilization to support implementation success (Slim et al. 2016).

### ERP item application in APHP and perspectives

As a whole, our results are disappointing when compared with the 80% high level (i.e. > 60%) ERP application observed in a large survey in orthopaedic surgery in the USA (Memtsoudis et al. 2020). However, this survey offers the first data on ERP application in APHP hospitals.

The definition we used for applied item of ERP (action traced in the patients’ chart and percentage of application >70%) can be considered as a high standard explaining potentially the final low application rate (28–44%) of EPR items across the three surgeries in 2017. However, this targeted level of appropriation is legitimate since it has been confirmed as a guarantee of clinically significant outcome improvement on adverse event and LOS (Gustafsson et al. 2011; Memtsoudis et al. 2020). Some ERP items were already applied in 2015 and comforted in 2017 such as non-invasive surgery for colectomy and hysterectomy, no colic preparation for colectomy, prevention of PONV and hypothermia, antibioprophylaxis, thromboprophylaxis for the three surgical models and regional anaesthesia for pain control for TKA. Most of those items refer to older specific recommendations such as those of the French Society of Anaesthesia and Intensive Care on antibioprophylaxis (Martin and Pourriat 1997), PONV (Diemunsch 2008), postoperative pain control (Aubrun et al. 2019) and thrombosis (Samama et al. 2011). This anteriority may explain why some of those items were already largely applied in 2015.

We observe a trend for improvement in ERP application in all orthopaedic and gynaecological departments. This improvement is reflected by a significant increase of ERP items’ implementation for 70% (12/17) of TKA ERP items and 44% for hysterectomy (7/16). However, important improvement beyond 70% of implementation is present for only 12% of ERP items for TKA (2/17) and hysterectomy (2/16) probably explaining the limited impact on LOS and complications. Since the top-down approach had no clear impact on ERP implementation, observed changes are probably due to natural evolution in time as suggested by similar evolution in control surgical models (i.e. THA, gastrectomy and ovariectomy). Interestingly in all individual data collected in our survey in 2015 and 2017, a significant correlation exists between the number of ERP items applied and LOS. After TKA and hysterectomy, LOS showed a negative relationship when more than 70% of ERP are applied. These results are in line with previous publication supporting that increased application of ERP protocols impact favourably LOS and certainly explain the slight reduction of LOS after TKA since this surgical model benefits of the largest improvement in ERP items in our survey (Memtsoudis et al. 2020).

Similar changes were not observed in visceral surgery where ERP application evolution appears heterogeneous in surgical department although this particular surgical model has been targeted for ERP for a long time (Gustafsson et al. 2011). For colectomy, the improvement is limited to only 24% (5/21) of ERP items with none reaching the 70% implementation target. Moreover, some important ERP items regress for colectomy between 2015 and 2017 (i.e. bowel preparation, immune-nutrition, intraoperative hemodynamic monitoring, intraoperative DXM). This underlines the difficulty not only to progress but also to maintain changes when they have been obtained (Ljungqvist et al. 2017). Sustainability on ERP application is based on continuous evaluation and quality improvement which was not operational in the hospitals evaluated in this survey. We have no more specific explanation on why colectomy model has less improvement than other surgical models but it will definitely become a priority in our institution.

This survey has clarified the level of practice for ERP and motivated future improvement which will be based this time mainly on implication of local health care provider multidisciplinary teams with institutional support (Ogunlayi and Britton 2017). The programme organized by Regional Health Agency (Agence Régionale de Santé) offering inter-institutional collaboration on ERP represents such combined initiative and it was installed in APHP after our top-down initiative. This strategy evolution started in 2017 in APHP will hopefully offer improvements for ERP in the future. This bottom-up approach will benefit from institutional support of APHP through support for ERP nurse appointment which appear determinant for implementation and sustainability of ERP (Gramlich et al. 2020).

### Strengths and limitations of the survey

This survey was able to collect a large sample of patients’ chart analysis in seventeen different university hospitals in APHP group. The direct analysis of chart allows measurement of effective medical performance. We respect the exhaustive description of all ERP items and outcomes (LOS and adverse events) in the six surgical model with clear definition of all data collected (Day et al. 2015). One limitation of this survey is related to its organization only in APHP, the largest University French hospital, limiting the generalization of the results. The other weakness is related to important reduction (38%) of data collection in 2017 due to impossibility of 6 hospitals out of 17 participants to collect data. Finally, the modifications proposed on 2016 to improve management of patient were proposed on a national (HAS recommendations, GRACE group affiliation) and institutional level (MOOC training), without personalized follow-up and coaching in each hospital involved in the survey.

### Conclusion

In this regional survey of 29 surgical departments in 17 university hospitals in APHP, a top-down approach did not significantly improve ERP application with significant reduction in LOS only for TKA surgery. Further improvement will require a more direct involvement of local health care providers backed up by institutional support.

## Data Availability

https://www.dropbox.com/s/ycbauj9i3zo09sh/URCPO_RAAC_RapportStatistique_LNM_V05.pdf?dl=0.
